# Metagenomics Reveals the Influence of Land Use and Rain on the Benthic Microbial Communities in a Tropical Urban Waterway

**DOI:** 10.1128/mSystems.00136-17

**Published:** 2018-06-05

**Authors:** Gourvendu Saxena, Suparna Mitra, Ezequiel M. Marzinelli, Chao Xie, Toh Jun Wei, Peter D. Steinberg, Rohan B. H. Williams, Staffan Kjelleberg, Federico M. Lauro, Sanjay Swarup

**Affiliations:** a Department of Biological Sciences, National University of Singapore, Singapore, Singapore; b Singapore Centre for Environmental Life Sciences Engineering (SCELSE), National University of Singapore, Singapore, Singapore; c Leeds Institute for Biomedical and Clinical Sciences, University of Leeds, Leeds, United Kingdom; d Singapore Centre for Environmental Life Sciences Engineering (SCELSE), Nanyang Technological University, Singapore, Singapore; e Centre for Marine Bio-Innovation, School of Biological, Earth and Environmental Sciences, The University of New South Wales, Sydney, Australia; f NUS Environmental and Research Institute, National University of Singapore, Singapore, Singapore; g Asian School of the Environment, Nanyang Technological University, Singapore, Singapore; h Synthetic Biology for Clinical and Technological Innovation, National University of Singapore, Singapore, Singapore; Hawaii Institute of Marine Biology

**Keywords:** benthic microbial communities, community composition and functions, land use, rain perturbations, urban environment

## Abstract

Growing demands for potable water have led to extensive reliance on waterways in tropical megacities. Attempts to manage these waterways in an environmentally sustainable way generally lack an understanding of microbial processes and how they are influenced by urban factors, such as land use and rain. Here, we describe the composition and functional potential of benthic microbial communities from an urban waterway network and analyze the effects of land use and rain perturbations on these communities. With a sequence depth of 3 billion reads from 48 samples, these metagenomes represent nearly full coverage of microbial communities. The predominant taxa in these waterways were *Nitrospira* and *Coleofasciculus*, indicating the presence of nitrogen and carbon fixation in this system. Gene functions from carbohydrate, protein, and nucleic acid metabolism suggest the presence of primary and secondary productivity in such nutrient-deficient systems. Comparison of microbial communities by land use type and rain showed that while there are significant differences in microbial communities in land use, differences due to rain perturbations were rain event specific. The more diverse microbial communities in the residential areas featured a higher abundance of reads assigned to genes related to community competition. However, the less diverse communities from industrial areas showed a higher abundance of reads assigned to specialized functions such as organic remediation. Finally, our study demonstrates that microbially diverse populations in well-managed waterways, where contaminant levels are within defined limits, are comparable to those in other relatively undisturbed freshwater systems.

**IMPORTANCE** Unravelling the microbial metagenomes of urban waterway sediments suggest that well-managed urban waterways have the potential to support diverse sedimentary microbial communities, similar to those of undisturbed natural freshwaters. Despite the fact that these urban waterways are well managed, our study shows that environmental pressures from land use and rain perturbations play a role in shaping the structure and functions of microbial communities in these waterways. We propose that although pulsed disturbances, such as rain perturbations, influence microbial communities, press disturbances, including land usage history, have a long-term and stronger influence on microbial communities. Our study found that the functions of microbial communities were less affected by environmental factors than the structure of microbial communities was, indicating that core microbial functions largely remain conserved in challenging environments.

## Abstract (precis)

Unravelling the microbial metagenomes of urban waterway sediments suggest that well-managed urban waterways have the potential to support diverse sedimentary microbial communities, similar to those of undisturbed natural freshwaters. Despite the fact that these urban waterways are well managed, our study shows that environmental pressures from land use and rain perturbations play a role in shaping the structure and functions of microbial communities in these waterways. We propose that although pulsed disturbances, such as rain perturbations, influence microbial communities, press disturbances, including land usage history, have a long-term and stronger influence on microbial communities. Our study found that the functions of microbial communities were less affected by environmental factors than the structure of microbial communities was, indicating that core microbial functions largely remain conserved in challenging environments.

## INTRODUCTION

Nearly two-thirds of global annual rainfall is at or near the equator (1). This zone is also the most populated part of the world, with 79% of the world population residing in tropical regions, mainly in urban settings (2). Metropolitan areas in the tropics, such as Singapore and Hong Kong, meet their growing demands for drinking water by engineering systems for capturing and redistributing storm water. These systems require the construction of extensive networks of paved drains and large canals (3, 4), the sustainable management of which is a challenging task, especially using ecological approaches. Water managers and urban planners around the world are implementing water-sensitive urban designs for sustainable management of waterways to maintain or enhance the self-cleaning potential of waterways. However, such practices lack understanding of microbial processes, underpinning the environmental services (5) provided by these ecosystems.

Sediment-associated microbial communities provide diverse ecological services and are the drivers of many biogeochemical processes in surface water systems (6, 7). Microbial communities are able to degrade a wide range of organic contaminants (8) where nutrient levels are low and available carbon sources are complex organic compounds (8). The ability to degrade complex organic contaminants for energy harvest is important for the survival of microbial communities in resource-deficient engineered urban waterways. These waterways are typically limited in nutrients and simple sugars but rich in complex organic compounds (9). As we move toward utilizing ecology-based approaches for sustainable water resource management, characterizing the composition and functions of benthic microbial communities and how they respond to urban factors, such as land use types and rainfall, becomes important.

Urban storm water in engineered waterways passes through many different land use types, such as residential and industrial areas. These areas contribute different loads of sediments, chemicals, and microbial communities, which together affect the ecosystem services of the engineered urban environment (10, 11) and downstream water collection reservoirs. The rain events lead to the removal of settled sedimentary microbial communities by resuspension and immigration of fresh populations of soil-associated microorganisms into the waterways. Therefore, factors such as land use types and rain could have major influences on the assembly and functioning of microbial communities in urban waterway systems. To the best of our knowledge, such influences have not been characterized for urban environments using deep sequencing metagenomic data.

**FIG 1:**
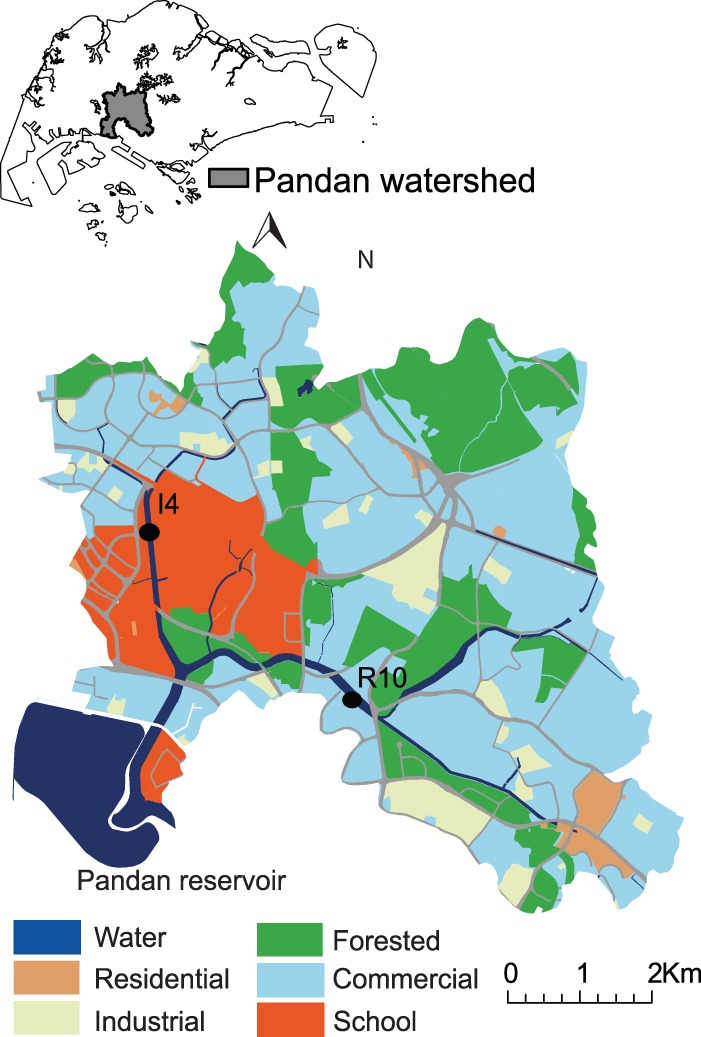
Model watershed and sampling locations. The inset shows Pandan watershed in the southwestern region of Singapore. The sampling locations were selected from representative and physically isolated residential and industrial land use types. (Adapted from reference 9 with permission of the publisher.)

In this study, we have examined a catchment in Singapore, a highly urbanized tropical city with two major land use types, residential and industrial, to identify the influence of land use and rain on the benthic microbial communities in urban waterways. These land use types covered >80% of the catchment (Fig. 1). We had previously found the microbial communities to be similar within the same land use type, despite being present in spatially nonconnected regions (9). While site I4 receives storm water and sediments exclusively from the eastern side of the waterway, which is an industrial land use type, site R10 receives storm water and sediments from residential land use type on both sides of the waterway (Fig. 1). Water and sediments at these sites do not cross-influence each other. We therefore, selected these sites for analyzing sedimentary microbiomes in urban waterways. As rain is one of the major perturbation factors in the tropics, we further sampled two rain events with similar intensity but of different durations and different dry periods preceding the event.

Here, we first describe the near complete coverage of species composition of the microbial communities of the catchment urban waterway system. To identify the influence of rain and land use on the microbial communities, we then compare the community composition and functions, as deduced from the metagenomic data for sediments of microbial communities, sediments before and after rain events, and sediments of the two land use types. We compare the urban waterway microbial communities identified from our study with those of other freshwater systems to assess the impact of low versus high contaminant levels on the diversity of microbial communities in urban waterways.

## RESULTS

### Urban catchment.

The catchment in this study covers an area of about 23 km^2^ and has two major land use types, residential and industrial (Fig. 1). The regions of residential land use type are more dispersed in the catchment, compared to the industrial areas, which has only one region (Fig. 1). The extensive subnetwork on drains and canals in this catchment consists of more than 16,000 m of big and intermediate canals (>20 m wide) and a dense network of small drains of length more than 300 km, which collect storm water from the local watershed and drain into Pandan reservoir. Therefore, the sedimentary microbial communities in the waterways reflect the microbial communities in the catchment. We collected sediment samples from six different locations at each land use site (Fig. 1) before and after rain across two rain events (see Fig. S1A in the supplemental material). This resulted in a total of 48 sediment samples.

10.1128/mSystems.00136-17.2 FIG S1  Sampling strategy and characteristics of the metagenome. (A) Sampling design for microbiome collection from Ulu-Pandan waterway. (B) Number of sequences for all samples combined. Please refer to Table S1 for detailed information. (C) The rarefaction curves were prepared by plotting the species hits against the number of randomly selected reads from the data matrix computed by repeatedly sampling the metagenomic reads from sample metagenomes and computing the number of species hits. Download FIG S1, TIF file, 2.8 MB. Copyright © 2018 Saxena et al. 2018 Saxena et al. This content is distributed under the terms of the Creative Commons Attribution 4.0 International license.

10.1128/mSystems.00136-17.5 TABLE S1  Details of the read statistics for sequencing and at each level of data processing. Download TABLE S1, DOCX file, 0.01 MB. Copyright © 2018 Saxena et al. 2018 Saxena et al. This content is distributed under the terms of the Creative Commons Attribution 4.0 International license.

### Environmental pressures and rain intensity.

**FIG 2:**
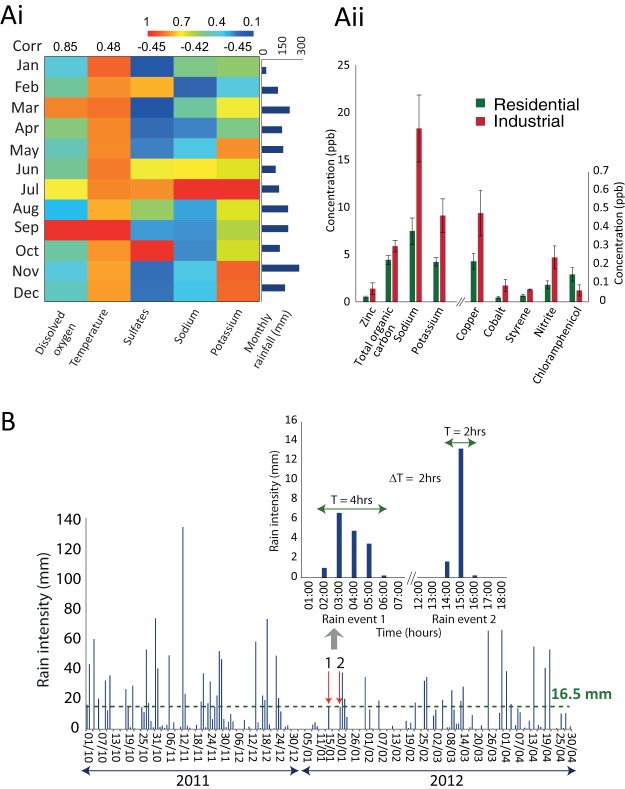
Environmental characteristics of the catchment. (Ai) Temporal variations of environmental variables, which showed significant correlations (Kendall tau, *P* < 0.05) with rain. (Aii) Significantly different (*P* < 0.05) environmental variables between the two land use types are shown. Error bars depict standard errors. (B) Distribution of daily rain events from October 2011 to April 2012. The inset graph shows the hourly rain intensity data for the two rain events.

In our previous work, we characterized 48 environmental parameters from the same catchment (9). Using this data, we established correlations between the rain intensity and environmental parameter matrix. The results revealed that dissolved oxygen and temperature have a positive correlation with the total suspended solids, while sodium and potassium display a negative correlation with the monthly rain intensity (*P* < 0.05) (Fig. 2Ai). Metals, such as copper, cobalt, and zinc, and organic contaminants, including styrene, were significantly higher in industrial areas than in residential areas (Fig. 2Aii).

In 2011 and 2012, the catchment received annual rainfalls of 3,423 mm and 2,700 mm with 190 and 170 rainy days and an average rainfall of 18 and 16 mm per rain event day, respectively (Fig. 2B). The rain data from October 2011 to April 2012 are shown in Fig. 2B. Our sampling in January falls in the northeast monsoon season (December-March), with relatively frequent dry-wet fluctuations. The rain intensities of rain event 1 (RE1) (15 January 2012) and rain event 2 (RE2) (20 January 2012) were both 16.25 mm. However, RE1 was of longer duration (4 h) than RE2 (2 h). The dry period prior to RE1 was about 20 days (more than 10-mm rain), while it was 5 days for RE2 (Fig. 2B).

### Microbial community composition in the urban waterway environment.

Sequencing of metagenomic DNA fragments using the Illumina HiSeq2000 platform resulted in 1,554,726,091 paired reads with a read length of 101 bp for the paired read or a total of 3,109,452,182 raw sequences (a total of 314,054,670,382 bp). Quality trimming to remove low-quality reads resulted in 3,023,688,898 (97.2%) reads (Fig. S1B). A total of 1,460,323,652 (48.3%) reads, with an average of 30,423,409 reads per sample (standard deviation, 91,74,804) were successfully mapped against the National Center for Biotechnology Information (NCBI) nonredundant (nr) protein database (12) (Fig. S1B). Rarefaction analysis of the 48 samples suggested that sequencing depth achieved a high level of saturation of the taxonomic richness, as all the plots reached asymptote, with the exception of one sample (Fig. S1C). Details of the read statistics for sequencing at each data processing stage are provided in Table S1.

**FIG 3:**
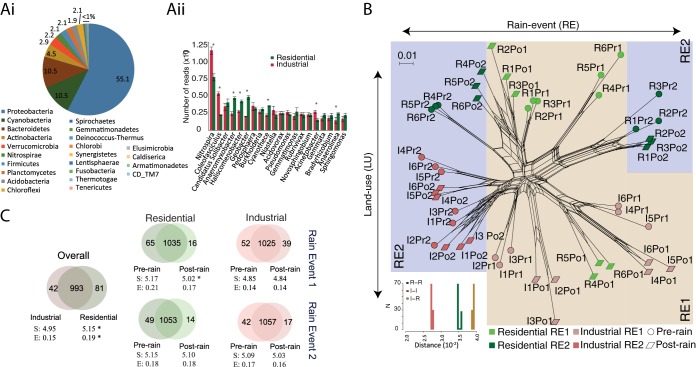
Structure of benthic microbial communities of residential and industrial land use types before and after two rain events in urban waterways are shown. (Ai) Relative abundances (as percentages) of benthic microbial communities at the phylum level in waterways. (Aii) Average abundance of top 20 bacterial genera, sorted based on abundance in the two land use types. Values that are significantly different (*P* < 0.05) in abundance in the two land use types are denoted by an asterisk. (B) The neighbor-net network of samples from the two land use types (industrial [red] and residential [green]) based on Bray-Curtis distance calculated using microbial community profiles at the species level. (Inset) Summary of distances within and between groups of microbiome profiles at the species level of samples from the two land use types. (C) Taxon distribution between the two land use types and pre- versus postrain for the two rain events. Lighter shades show differential genera (*t* test; *P* < 0.05, standard Bonferroni correction, permutations = 999) and darker shades show commonly occurring genera. Diversity indices are shown as follows: S, Shannon index (entropy); E, Buzas and Gibson’s evenness (*, *P* < 0.05).

The combined metagenome of benthic microbial communities in urban waterways was composed of 75 phyla and 4,163 species, based on reads mapped to NCBI nr database. There were 1,116 genera identified that could be discriminated with a higher level of confidence. More than 99% of the mapped reads in the sediments of urban waterways were represented by 23 phyla (~30%) (Fig. 3Ai). Almost 80% of the annotated metagenome reads belonged to the top four phyla: *Proteobacteria*, *Cyanobacteria*, *Bacteroidetes*, and Actinobacteria. Hierarchical clustering showed different trends for the taxonomic groups. Four of these clusters (Fig. S2) can be explained by factors such as land use or rain events. Table S2A lists the top 10 genera in these clusters. Clusters 1, 3, and 4 represent taxonomic groups that were enriched in either land use type. This group showed high abundance of genera involved in N-S cycling, such as *Nitrosospira*, *Nitrosovibrio*, *Nitrosopumilus*, *Beggiatoa*, and *Sulfuritalea*. Cluster 2 represents taxonomic groups that appeared only after the rain event. These include reads from viruses with diverse hosts such as *Asfivirus* and *Phaeovirus*, the oral cavity commensal *Alloprevotella*, and the deep ocean genus *Zunongwangia*.

10.1128/mSystems.00136-17.3 FIG S2  Hierarchical clustering of taxa profiles at the genus level. Genus present in at least 50% of samples were used in this analysis. The yellow boxes show distinct patterns of taxon abundances. Please refer to Table S2A for the list of genera. Download FIG S2, TIF file, 2.9 MB. Copyright © 2018 Saxena et al. 2018 Saxena et al. This content is distributed under the terms of the Creative Commons Attribution 4.0 International license.

10.1128/mSystems.00136-17.6 TABLE S2  (A) List of top 10 genera in each cluster, filtered based on significance and sorted based on abundance in the boxed clusters of Fig. S2. (B) List of top 20 functions, filtered based on significance and sorted based on abundance in the boxed clusters of Fig. S3. Download TABLE S2, DOCX file, 0.01 MB. Copyright © 2018 Saxena et al. 2018 Saxena et al. This content is distributed under the terms of the Creative Commons Attribution 4.0 International license.

### Functional potential of microbial communities.

Microbial communities of urban waterways had a high abundance of genes related to carbon metabolism, which was significantly higher in residential areas (*P* < 0.05) than in the industrial areas (Fig. S3A), based on *t* test with standard Bonferroni corrected *P* values. The second highest abundant genes were genes related to virulence factors and protein and nucleic acid metabolism (Fig. S3A). There was no significant difference between the two land use types for other gene categories, except metabolism of aromatic compounds (high abundance in residential areas; *P* < 0.05), membrane transport (high abundance in residential areas; *P* < 0.01), sulfur metabolism (high abundance in residential areas; *P* < 0.01), and photosynthesis (high abundance in industrial areas; *P* < 0.01) (Fig. S3A). Different trends of functional gene abundance were evident from the hierarchical clustering results. Three of these clusters (Fig. S3B) are explained by factors such as land use or rain events. Table S2B lists the top 10 functional genes in these clusters, based on abundance. Cluster 1 largely represents functional genes enriched in the microbial communities from RE1. The genes are involved in biosynthesis, e.g., biosyntheses of flavonoids and carotenoids, organic remediation, such as xylene degradation, and secretion, such as bacterial secretion system. Cluster 2 showed functions enriched mainly in industrial samples, which included functions related to the infection process of pathogens.

10.1128/mSystems.00136-17.4 FIG S3  Distribution of microbial functions in urban waterways. (A) Major functional groups distributed in residential and industrial land use. Significance was tested using *t* test (*, *P* < 0.05; **, *P* < 0.01). (B) Hierarchical clustering of functional genes. Functions present in at least 50% of samples were used in this analysis. The yellow boxes show two distinct patterns of functional gene profiles. Please refer to Table S2B for the list of functional genes. Download FIG S3, TIF file, 2.8 MB. Copyright © 2018 Saxena et al. 2018 Saxena et al. This content is distributed under the terms of the Creative Commons Attribution 4.0 International license.

### Influence of land use on microbial communities.

The microbial communities from the two land use types were clearly separated when analyzed via neighbor-net networks from metagenomic DNA sequences and MEGAN taxonomic annotation (Fig. 3B). To further test the network robustness, a read-level bootstrapping of network clustering was conducted with 100 iterations from random subsamples of the data sets as described in Materials and Methods. The residential and industrial samples were clearly and reproducibly separated, with the residential group showing a greater within-group variation relative to the industrial samples (Fig. 3B).

At the genus level, most reads were from *Nitrospirae* (*Nitrospira*) and *Cyanobacteria* (*Coleofasciculus*) (Fig. 3Aii). The reads for these taxonomic groups, which are involved in nitrogen cycling and carbon fixation, were more abundant in industrial areas than in residential areas (*P* < 0.05 by *t* test) (Fig. 3Aii). Reads from genera with low abundance in the catchment, such as “*Candidatus* Solibacter,” *Anaeromyxobacter*, *Haliscomenobacter*, *Geobacter*, and *Anaerolinea* represented a higher relative proportion in the community from residential areas (Fig. 3Aii). The distribution of taxonomic groups (genera) in different samples suggested that the benthic microbial communities in residential land use were more diverse and dispersed than in industrial sediments (*P* < 0.05 by *t* test) (Fig. 3C). The distribution of taxonomic groups (genera) provided further evidence that the benthic microbial communities in residential land use were more diverse, as the number of unique taxa was greater in residential sediments with approximately twice as many genera in residential sediments (81 genera) than in industrial sediments (42 genera) (Fig. 3C).

These results were further substantiated by permutational multivariate analysis of variance (PERMANOVA) analyses (Table S3A). The influence of land use was more dominant than rain perturbation on the structure of microbial communities. Pairwise results for 16S rRNA-based taxonomic profiles showed communities from both land use types were significantly different in both rain events (Fig. 3B; see Table S3A also). Permutational multivariate analyses of DISPersion (PERMDISP) analysis identified significant dispersion between the microbial communities from residential and industrial land use types (Table S4).

10.1128/mSystems.00136-17.7 TABLE S3  PERMANOVAs based on Bray-Curtis (BC) similarity measure for square root-transformed data (abundances) of (A) taxonomic profiles generated using 16S rRNA gene sequences and (B) functional genes/pathways using SEED databases (level 3). Land use type (LU) was fixed with two levels (industrial [I] and residential [R]), before/after rain (B/A) was fixed and orthogonal, with two levels (before versus after), rain event (RE) was random and orthogonal, with two levels. The replicates were the sediment samples (*n* = 6). *P* values were calculated using 9,999 permutations under a reduced model. Nonsignificant interactions with *P* > 0.25 were pooled. Download TABLE S3, DOCX file, 0.01 MB. Copyright © 2018 Saxena et al. 2018 Saxena et al. This content is distributed under the terms of the Creative Commons Attribution 4.0 International license.

10.1128/mSystems.00136-17.8 TABLE S4  PERMDISP analyses based on Bray-Curtis (BC) similarity measure for square root (sqrt)-transformed abundances or Jaccard similarity measure (composition; presence/absence) of genomic DNA (gDNA) HiSeq at the genus level (A) and functional gene abundances (B). *P* values were calculated using 9,999 permutations. Download TABLE S4, DOCX file, 0.01 MB. Copyright © 2018 Saxena et al. 2018 Saxena et al. This content is distributed under the terms of the Creative Commons Attribution 4.0 International license.

**FIG 4:**
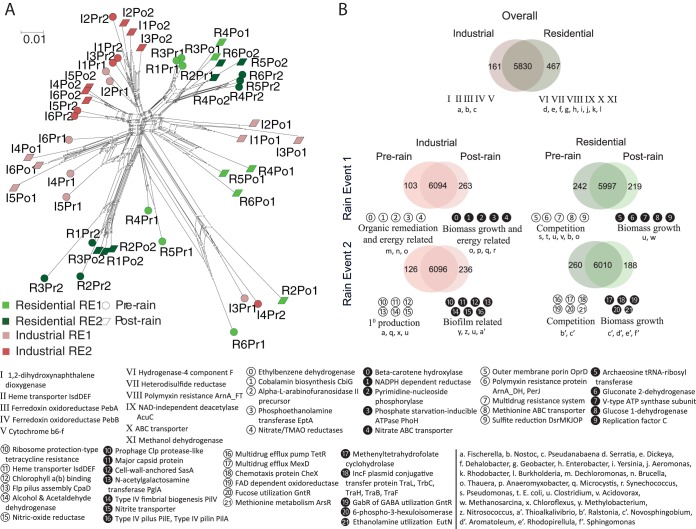
Distribution of microbial functions in urban waterways. (A) The neighbor-net network cluster of samples from the two land use types based on Bray-Curtis distance calculated using microbial community functional potential annotated through SEED databases. (B) Functional gene distribution between the two land use types and pre- versus postrain for the two rain events. Lighter shades show differential genera (*t* test; *P* < 0.05, standard Bonferroni correction, permutations = 999) and darker shades show commonly occurring genera. Functional gene categories are defined by most abundant functional genes. The functional genes in respective categories are annotated with numbers. Taxonomy assignments of genes in different groups are shown as lowercase letters.

The distribution of functional gene abundances also showed clear separation between the two land use types (Fig. 4A). The number of unique functions was greater in residential sediments, with approximately four times as many functional genes (467 genes) compared to industrial sediments (161 genes) (Fig. 4B). The most abundant and significantly different functional genes between the two land use types were engaged in organic remediation, such as 1,2-dihydroxynaphthalene dioxygenase and energy processes, including heme transporter IsdDEF and ferredoxin oxidoreductase PebA in industrial areas, mostly from cyanobacteria. In residential areas, of the significantly different functional genes, the most abundant were related to competition, such as polymyxin resistance (ArnA) and energy processes, including hydrogenase-4 component F, heterodisulfide reductase, and NAD-independent deacetylase AcuC (Fig. 4B).

These results were further substantiated by PERMANOVA analyses (Table S3B), where the influence of land use was more dominant than rain perturbation on the structure of microbial communities.

### Influence of rain on microbial communities.

RE1 mediated a significant difference in the pre- versus postrain microbial community diversity in residential land use sediments (Fig. 3C). Moreover, the diversity of microbial communities increased significantly (*P* < 0.05 by *t* test) from post-RE1 to the pre-RE2 in both land use types (Fig. 3C). The prerain sediment microbial communities had more unique genera than the postrain sediment microbial communities, with slightly reduced numbers in RE2 (Fig. 3C). The functions of the microbial communities differed significantly before and after rain only during RE1 (4-h duration), compared to RE2 (2-h duration) (Fig. 1) (PERMANOVA; Table S3). There was no difference in dispersion between pre- and postrain microbial functions (PERMDISP; Table S4).

Functions of microbial communities within both land use types showed inverse trends before and after rain perturbations. In residential benthic microbial communities, the unique functions decreased after rain events, while in industrial areas, their numbers increased after the rain events (Fig. 4B). The profiles of differential functions (before and after rain) remained similar for the two rain events in the benthic microbial communities of residential land use types. The majority of the most abundant functional genes (three out of five) in residential microbial communities belonged to functions associated with competition, such as outer membrane porins, polymyxin and multidrug resistance prerain, and were replaced by genes belonging to biomass growth, such as tRNA-ribosyl transferase and methenyltetrahydrofolate cyclohydrolase in postrain microbial communities (Fig. 4B). Despite the similarities in the functional potential across the two events, they were derived from different sets of taxonomic groups (Fig. 4B). In the industrial benthic microbial communities, the most abundant function of those that differed before and after rain changed from organic remediation, such as ethylbenzene dehydrogenase, biomass growth, including phosphoethanolamine transferase and nitrate reductase to β-carotene hydroxylase and phosphate starvation-inducible ATPase during RE1 (Fig. 4B). During RE2, the majority of the most abundant functional genes before rain were related to primary production, such as heme transporter IsdDEF and chlorophyll *a*(*b*) binding. These genes were replaced by viral marker genes and biofilm-related genes, such as prophage clp protease, major capsid protein, and the type IV pilus (PilE) (Fig. 4B). As for the residential areas, the functional genes identified in the two rain events were attributed to different sets of taxonomic groups. Functions for genes from certain genera, such as *Thauera* and *Novosphingobium*, were related to organic remediation, while *Clostridium* contained genes involved in carbon and nitrogen fixation distributed between land use types and the two rain events (Fig. 4B).

### Benthic community structure is similar to undisturbed freshwater systems.

**FIG 5:**
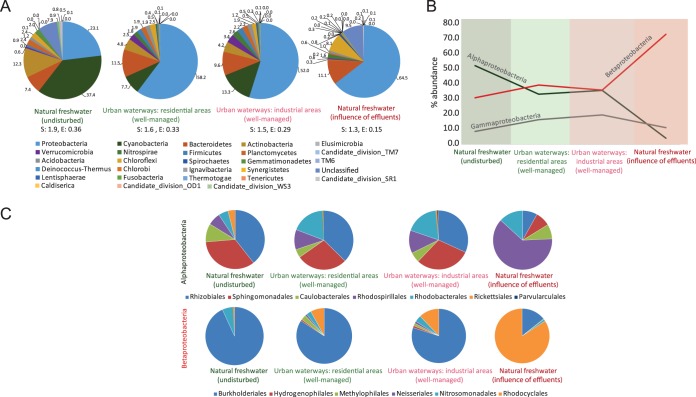
Taxon distribution in sediment microbial communities of natural river and urban waterway systems. (A) Comparison of phylum distribution of the two benthic microbial communities in river water system (13) with urban water system at the phylum level. Diversity indices are shown as follows: S, Shannon index (entropy); E, Buzas and Gibson’s evenness (Mann-Whitney U test, *n* = 3, *P* < 0.001). (B) Trends of different classes in the *Proteobacteria* phylum between the three microbial communities. (C) Distribution of different orders in the *Alpha*- and* Betaproteobacteria* classes of the *Proteobacteria* phylum in the three microbial communities.

We then compared the microbial communities from urban waterways with that of a natural freshwater system (river) published elsewhere (13). The samples in that study were collected from a pristine site of the river (upstream of urban influence) and a site influenced by industrial effluents on the same river (downstream of urban influence) (13). Diversity analysis of upstream and downstream sedimentary microbial communities (13) revealed that benthic microbial community profiles decreased in diversity values with increased exposure to urban influences. The pristine site (13) had the most diverse microbial communities, followed by the microbial communities of both land use types in urban waterways of the present study. The microbial communities from the site influenced by industrial effluents had the least diversity compared to the pristine site and urban waterways (*P <* 0.001 by Mann-Whitney U test; *n* = 3) (Fig. 5A). The abundance of primary producers, such as cyanobacteria, which is highest at the pristine site of the river (37%), was maintained in the waterway system (10%) of our study. However, primary producers were completely outnumbered in the downstream system (Fig. 5A). The differences in the relative abundance of the top five phyla in the natural freshwater system and our study show that genera involved in primary productivity (*Cyanobacteria*) and carbon cycling (*Actinobacteria*) are reduced in microbial communities from urban influenced sites, whereas heterotrophs (including *Proteobacteria*, *Bacteroidetes*, and *Verrucomicrobia*) increase in abundance (Fig. 5A). Within the *Proteobacteria*, the abundance of *Alphaproteobacteria* was reduced and the abundance of *Betaproteobacteria* increased in microbial communities under urban influence (Fig. 5B). However, the benthic microbial communities in the urban waterway system maintained the structure of *Alpha*- and *Betaproteobacteria* close to the benthic microbial communities of undisturbed natural freshwater (Fig. 5C).

## DISCUSSION

To understand the influence of urban factors, such as land use, and rain on the benthic microbial communities in a tropical urban waterway, we focused on an ~25-km^2^ catchment area that encompasses two major land use types before and after rain events. As sediments from eroding soil enter the waterways after rain events, the sedimentary microbial communities are exposed to multiple stress factors. These factors include land use-specific chemical stressors (14–16) and different shear forces resulting from increased flow after rain (17–19), which are dependent on both sediment and rain properties. The current experimental design allowed us to study the influence of the above two factors, namely, land use types and rain on the benthic microbial communities. By adopting next-generation sequencing (NGS), we sequenced the metagenome to near complete coverage of species, and detected and identified nearly all microbial species, annotated in the NCBI nonredundant database, of the benthic microbial communities in this urban waterway (see Fig. S1 for nearly saturated rarefaction curve for all samples). The microbial communities in the urban waterways are very complex (rarefaction curve saturation [Fig. S1C] with <50% hits in the database [Fig. S1B]), and we have identified almost all the species possible using the current databases.

The benthic microbial communities contained 75 different phyla, of which 23 dominated the communities. We identified the taxonomic groups responsible for small, yet significant differences in the microbial communities between pre- and post-rain events for the two land use types (Fig. 3C). The presence of *Alphaproteobacteria*, *Cyanobacteria*, and *Actinobacteria* in freshwater systems of a highly urbanized city like Singapore is significant, as these taxa are highly sensitive to urban pressures, such as organic compounds and antibiotics (13, 20). Members of the microbial communities in the residential areas were more diverse (Shannon’s index; *P* < 0.05) and even (Buzas and Gibson’s evenness; *P* < 0.05) in microbial community structure than those in industrial areas (Fig. 3C). In particular, a higher percentage of *Coleofasciculus* (*Cyanobacteria*)- and cyanobacterium-associated primary productivity genes were observed in the industrial samples. This possibly results from the lower concentrations of antibiotics, such as chloramphenicol, observed in the industrial areas (9), as most cyanobacteria have been shown to be highly sensitive to antibiotics (20). The larger amounts of organic contaminants, such as styrene, could be stimulating the growth of xenobiotic-degrading strains of *Anaeromyxobacter*, *Geobacter*, and *Anaerolinea* in industrial areas (21, 22). Therefore, there is further scope for reducing the concentrations of antibiotics and organic contaminants in residential and industrial areas. Conditions favoring nitrite oxidation, such as the higher availability of nitrite coupled to a lower concentration of nitrate (9), might explain the proliferation of *Nitrospira* species in industrial areas. Certain *Nitrospira* species can utilize the complete nitrate reduction to ammonium via nitrite (23), which may account for the higher abundance of *Nitrospira* in the urban waterways of this study.

Increase in the microbial diversity after rain event 1 (RE1) and before rain event 2 (RE2) can be attributed to the higher availability of dissolved oxygen in water during the wet period. Increased temperature during the wet period, as shown by the positive correlation of water temperature with rain, might be due to the heated island effect (24). Storm water appears to mitigate the heated island effect but at the same time exposes the microbial communities in waterways to elevated temperature. Therefore, it should be taken into consideration while planning to dissipate land surface heat using rain water. A high abundance of heterotrophy- and biomass growth-related genes in the microbial communities after rain reflects increased dissolved oxygen due to frequent rain events in the catchment, while the presence of organic remediation genes before rain appears to be linked to low levels of dissolved oxygen. Hence, microbial communities are responding to small changes in environmental conditions through specific functional pathways, which can be exploited to implement specific management interventions at the source to enhance the self water-cleaning potential waterways. Overall, the study found a clear separation between the metagenomic profiles of the microbial communities belonging to residential and industrial sediment types, both taxonomically and functionally. These results are consistent with studies of microbial community structure and functions in comparable systems, such as oil-polluted environments (25), river systems with inputs from local land uses (16, 26), and natural vegetation environments (27). The microbial communities in such environments showed adaptation to the local environmental conditions both at the taxonomic and functional levels.

As this is the first comprehensive urban benthic microbial community metagenomic study, a comparison with information derived from other freshwater systems may address whether urbanization exerts selective pressures that reduce microbial community diversity. Such a relationship was observed in Nanxijiang River near Wenzhou city in the Zhejiang Province of China (13). The microbial communities of the urban water system studied here had Shannon diversity and Buzas and Gibson’s evenness indices similar to those from undisturbed Nanxijiang River communities, upstream of an urbanized area. The diversity indices of the urban waterway microbial communities were significantly higher than that of microbial communities from industrially influenced location of the same river. Such patterns suggests that well-managed waterways, that keep the contaminants levels below the permitted limits, can support microbial communities with a diversity and evenness comparable to those of undisturbed natural freshwater systems. Such diverse microbial communities can provide a wide range of ecological services and enhance the self water-cleaning capacity of waterways.

Reads mapped to diverse categories of amino acid and carbohydrate metabolism genes were highly abundant in the microbial communities of the waterways, supporting the notion of a large gene pool for utilizing a wide range of carbon sources. The existence of a flexible gene pool and its underlying phylogenetic structure is also supported by the high multivariate dispersion in the taxonomic analysis. The higher abundance of functional genes related to competition prior to rain events suggests that competition is driving the higher microbial community diversity in residential areas (Fig. 4B). The majority of microbial functions in the prerain residential sedimentary microbial communities have high abundance of, for example, outer membrane porin OprD, polymyxin resistance protein and multidrug resistance systems, which can be classified as genes providing a competitive advantage. For example, OprD provides competitive advantage to taxon groups through efficient uptake of nutrients (28). Likewise, polymyxin resistance protein (29) and multidrug resistance systems (30) provide a competitive advantage to taxonomic groups by either neutralizing antibiotics or by metabolizing the drugs as nutrients in such nutrient-deficient systems. In contrast, the functions of microbial communities in industrial areas are more influenced by environmental pressures (anthropogenic chemicals), rather than within-community competition, as suggested by the high abundance of ethylbenzene dehydrogenase gene (a common hydrocarbon-oxidizing enzyme). In summary, in urban waterways, the less diverse microbial communities might result from external environmental selection pressures, while the more diverse microbial communities are influenced by intracommunity competition.

Significant correlation between the taxonomic and functional profiles suggests that the functional potential may not be entirely independent of the structure of the microbial communities (Table S5). The observed shifts in the functional potential in response to perturbations, therefore, appear to be a result of changes in the relative abundances of taxonomic groups with some degree of functional redundancy rather than selection of more favorable adaptive traits.

10.1128/mSystems.00136-17.9 TABLE S5  Outcome of individual RELATE tests: Spearman’s correlation coefficients (ρ) for relationships between taxa and function based on Bray-Curtis similarity measure for square root-transformed abundances of taxa (genus level) or functional genes/pathways. All correlations were significant with *P* < 0.0004. *P* values were calculated using 9,999 permutations. Download TABLE S5, DOCX file, 0.01 MB. Copyright © 2018 Saxena et al. 2018 Saxena et al. This content is distributed under the terms of the Creative Commons Attribution 4.0 International license.

With increasing urbanization and changing climate, this study provides insights into the ability of microbial communities to maintain diversity in a manner comparable to those of the natural freshwater systems, in response to pressures associated with land use, rain, and management practices. Further, our study will also advance the development of management practices that benefit from ecologically based approaches to improve surface water quality. Finally, it provides a microbial metagenomic data set from urban waterway sediments that can serve as a reference for microbial ecology studies pertaining to urban environments.

## MATERIALS AND METHODS

### Sampling design.

This study was designed to capture the taxonomic and functional diversity of microbial communities in urban waterways and the influence of perturbation by rainfall events and land use pressures on these communities. This study was conducted in the Ulu-Pandan catchment in the southwestern region of Singapore and drains water into the Pandan reservoir (Fig. 1). Sampling locations represent a subset of all industrial and residential locations sampled in a previous study that used microarray-based technologies and encompassed a wide range of environmental variables associated with changes in microbial communities (9).

Sediment samples were collected from the top 1-cm layer of sediments from the drains at an industrial (I) location (1.3356° N, 103.7491° E) and a residential (R) location (1.3188° N, 103.7708° E; Fig. 1) before and after rain during two independent rain events (Fig. 2B). Samples were collected about 1 to 2 h before rain (pre) and 3 to 4 h after rain (post), with a time lag of about 30 min between sampling at the two locations (Fig. 1). We followed the 2-h weather forecast provided by the National Environment Agency of Singapore (http://www.nea.gov.sg/weather-climate/forecasts/2-hour-nowcast) to guide our sampling. Samples were collected during the two rain events: 15 January 2012 (rain event 1 [RE1]) and 20 January 2012 (rain event 2 [RE2]) (Fig. 2B).

For each combination of timing (before rain versus after rain), rain event, and location, six independent sediment samples were collected (total of 48 samples; Fig. 2A) from the side drain of the canal, which collects the storm water from the local industrial or residential areas. It is worth noting that although the industrial site sampled in this study is close to a residential area on the west, the water and sediment that it receives comes from the highly industrial eastern area (Fig. 1). Each sample was prepared by pooling sediment samples from five random sites within a 5-m diameter and collected in clean nuclease-free Falcon 50-ml conical centrifuge tubes (Thermo Fisher Scientific) using an autoclaved spatula. The 50-ml tubes were fully filled with the sediments, with about 5 ml of pore-water added. The samples were immediately transferred to an icebox and transported to the laboratory for DNA extraction. The time elapsed from the collection of the first sample to the beginning of DNA extraction was <2.5 h.

### Rain data.

The National University of Singapore (NUS) weather station, which is maintained by the Department of Geography, collects real-time meteorological data at 1-h intervals. Hourly rain intensity data for the years 2011 and 2012 were processed to obtain total rain intensity per day.

### DNA extraction and cleanup.

Sediment samples were thoroughly mixed using a clean spatula prior to weighing. Sediments mixed with water were siphoned into 2-ml tubes and centrifuged to discard the pore-water. Sediments were then weighed to 200 ± 20 mg for extracting DNA. DNA extraction was performed using the FastDNA spin kit for soil (MP Biomedicals, USA) according to the manufacturer’s protocol. The purified DNA was eluted in nuclease-free water and cleaned using OneStep PCR inhibitor removal kit (Zymo Research Corporation, USA) following the manufacturer’s protocol. DNA quantity was quantified using a NanoDrop spectrophotometer (Thermo Scientific) and Quant-iT PicoGreen double-stranded DNA (dsDNA) assay kit (Invitrogen) according to the manufacturer’s protocol. Samples were stored in −80°C until further processing.

### Illumina library preparation and sequencing for sediment samples.

Prior to library preparation, the quality of the DNA samples was assessed on a Bioanalyzer 2100, using a DNA 12000 chip (Agilent). Sample quantitation was performed using Quant-iT PicoGreen dsDNA assay kit (Invitrogen). Next-generation sequencing library preparation was performed following Illumina’s TruSeq DNA sample preparation protocol with the following modifications: to reduce the concentration of substances in the extracted DNA samples that may inhibit library preparation reactions, each DNA sample was purified with the OneStep PCR inhibitor removal kit (Zymo Research Corporation, USA) prior to library preparation. The purified DNA samples were then sheared on a Covaris S220 system to ~500 bp (samples 31 to 44) and ~300 bp (samples 291 to 324) (see Table S1 in the supplemental material), following the manufacturer’s recommendation. Size selection was performed on a Sage Science Pippin Prep instrument, using a 2% ethidium bromide agarose cassette and selecting for a tight spectral peak around 600 bp and 400 bp for samples 31 to 44 and samples 291 to 324, respectively. Each library was tagged with one of Illumina’s TruSeq LT DNA barcodes to allow for library pooling prior to sequencing.

Invitrogen’s Picogreen assay was used for library quantitation, and the average library size was determined by running the libraries on a Bioanalyzer DNA 7500 chip (Agilent). Library concentrations were normalized to 4 nM and validated by quantitative PCR (qPCR) on a StepOne Plus real-time thermocycler (Applied Biosystems), using qPCR primers recommended in Illumina’s qPCR protocol, and Illumina’s PhiX control library as a standard. Libraries were then combined into four pools, and each pool was sequenced in two lanes of an Illumina HiSeq2000 sequencing run at a read length of 101 bp for each paired end.

### Data processing and analyses.

Raw sequence reads for all 48 samples were quality trimmed/adapter clipped using cutadapt-1.2.1 (31) with default parameters (except for -m 30 -q 20). The quality-trimmed reads from all the samples were searched against the National Center for Biotechnology Information (NCBI) nonredundant protein database (version 30.07.2012) (12), using RapSearch2 (32). Details of read statistics for all samples are summarized in Table S1. The data matrix for sample-based rarefaction curves were computed using the vegan community ecology package (33) by repeatedly sampling the metagenomic reads from sample metagenomes and computing the number of leaves to which taxa (species level) were assigned (i.e., uncollapsing the taxonomy tree to the species level). The rarefaction curves were then prepared by plotting the taxonomy hits at the species level against the number of reads selected randomly. “Leaves” are taxonomic nodes annotated at a given taxonomic level (species in our case).

### Metagenome-based taxonomic annotation.

We used MEtaGenome ANalyzer (MEGAN) to analyze the taxonomic profiles of 48 sediment samples collected from two land use types. The resulting output files of paired read sequences were imported and analyzed using the paired-end protocol of MEGAN (34) to obtain taxonomic profiles using the lowest common ancestor (LCA) algorithm. When processing the RapSearch2 output files by using MEGAN, we used parameter settings of “Min Score = 35,” “Top Percent = 10,” “Min Support = 25” and “Minimum sequence complexity threshold = 0.44,” as a threshold for species mapping, to create MEGAN-proprietary “rma files” (hereon referred as RMAFs). Reads that did not have any match to the respective database were placed under the “no hits” node.

### 16S rRNA-based taxonomic annotation.

The 16S rRNA-based taxonomic profiling was conducted using an in-house-developed pipeline, RiboTagger (35). The metagenomic DNA reads were scanned for 33-nucleotide tag sequences from the V6 region as previously described (35, 36). Briefly, a universal primer (CGACRRCCATGCANCACCT) for the 16S rRNA hypervariable region (V6) was used to scan each sequencing read to obtain 33-nucleotide sequences downstream of the primer, and the 33-nucleotide sequences were defined as the V6 tag of the 16S rRNA gene. Short reads that did not cover the full 33-nucleotide region were discarded. The universal primer matches 94% of 16S rRNA sequences in the Ribosomal Database project (RDP) database (37). Each V6 tag was then used as a signature sequence to represent one operational taxonomic unit.

### Functional annotation.

Annotated taxonomic assignments were linked to functional roles with Kyoto Encyclopedia of Genes and Genomes (KEGG) (38) classifications using MEGAN.

### Multiple metagenome comparisons.

The output from MEGAN was normalized to the smallest data set size to allow between-sample comparison of taxonomic abundances for all samples. Comparative abundance of read counts at different levels of NCBI taxonomy (“class,” “order,” “family,” “genus,” and “species”) and functional categories (KEGG) were exported from all sample comparison files for later statistical analyses. We computed unrooted networks from Bray-Curtis distance matrices and a neighbor-net algorithm (39) for different levels of comparative taxonomic and functional profiles as previously described (40).

### Statistical analysis.

In order to adjust for differences in sequencing depth among the 48 samples, we subsampled six million reads with replacement from the genus-level annotations from each of the 48 data sets. We then computed an unrooted phylogenetic network with SplitsTree4 (41) using the Bray-Curtis distance metric. This process was repeated 100 times, generating 100 bootstrapped weighted-edge networks. The total number of nodes in these networks ranged from 707 to 1,273 (mean of 868). To quantify the degree of reproducibility of these networks, we first calculated the shortest path matrix for all nodes and then defined the submatrix of the shortest path matrix defined by the 48 sample nodes. Within- and between-group summary distances were subsequently computed using the methods of Gower and Krzanowski (42). All computations were performed using R version 3.0.2 (43).

Taxonomic and functional profiles (normalized read counts of taxa or orthologous genes exported from MEGAN) of the microbial community were compared between both land use types (industrial versus residential) and rain perturbation (“pre” versus “post” rain) using the permutational multivariate analysis of variance (PERMANOVA) (44) add-on in Plymouth Routines In Multivariate Ecological Research (PRIMER-v6) (45). For such analyses, we considered the taxonomic profile of the full data set at the genus level and functional profiles (KEGG levels 2 and 3). Each data set was square root transformed prior to calculating a Bray-Curtis similarity matrix. Similarity percentage analysis (SIMPER) analyses (46) were used to determine which taxon/function contributed most to the observed differences. In addition, dispersion (variability) of the multivariate data was compared between land use types and before versus after rain using permutational multivariate analyses of DISPersion (PERMDISP) (45). Finally, “relate analysis” was used to determine relationships between taxon and function similarity matrices (47). The significantly different taxonomic groups, functional genes, and differences in the diversity indices are calculated based on *t* test with standard Bonferroni correction and 999 permutations. The significance of the differences between the diversities of microbial communities from urban waterways and microbial communities from a previously published study of Nanxijiang River near Wenzhou city in the Zhejiang Province of China (13) were computed using Mann-Whitney U test.

### Data availability.

The metagenome sequencing data sets supporting the conclusions of this article are available in the NCBI Sequence Read Archive (SRA) study under accession number SRP051069 and Study BioProject number PRJNA267173.

10.1128/mSystems.00136-17.1 TEXT S1 Explanation and formula used to calculate Shannon’s diversity index and Buzas and Gibson’s evenness for the composition and functions of sedimentary microbial communities. Download TEXT S1, DOCX file, 0.01 MB. Copyright © 2018 Saxena et al. 2018 Saxena et al. This content is distributed under the terms of the Creative Commons Attribution 4.0 International license.
