# Supplementary figures and images for: Metagenomics Reveals the Influence of Land Use and Rain on the Benthic Microbial Communities in a Tropical Urban Waterway
Source: mSystems. 2018 Jun 5;3(3):e00136-17. doi: 10.1128/mSystems.00136-17 (PMC5989131; doi:10.1128/mSystems.00136-17)

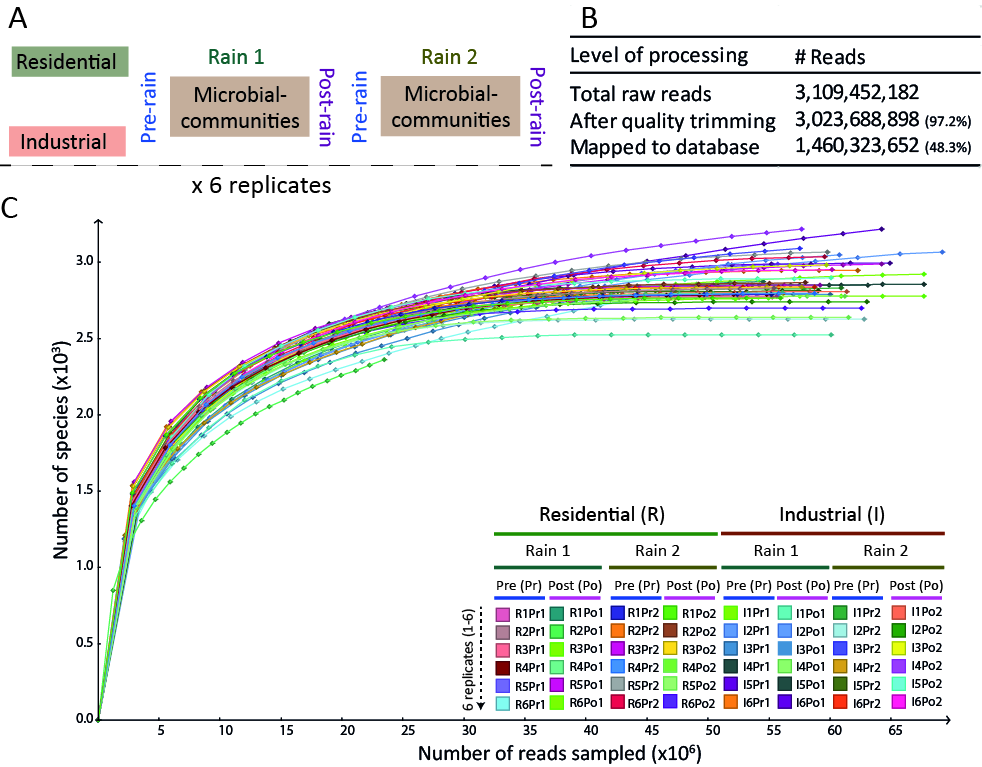

Supplement: FIG S1 [file sys003182236sf1.tif]

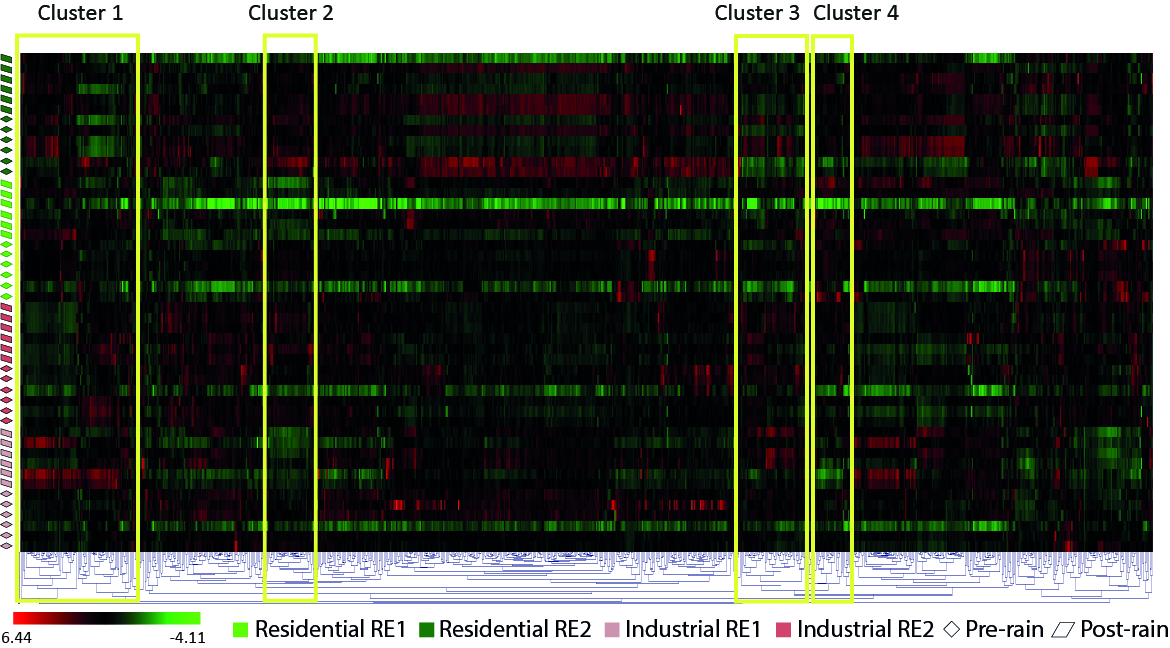

Supplement: FIG S2 [file sys003182236sf2.tif]

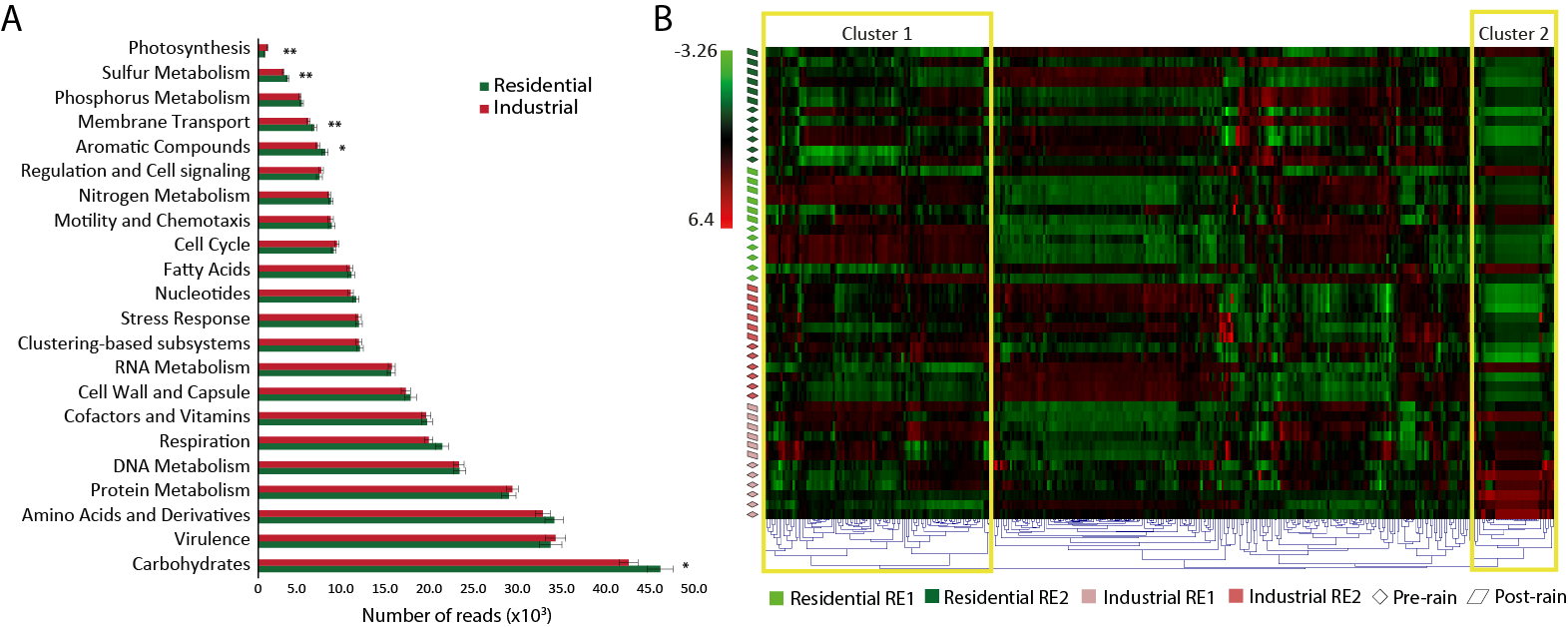

Supplement: FIG S3 [file sys003182236sf3.tif]
