# Supplementary material for: Metagenomics Reveals the Influence of Land Use and Rain on the Benthic Microbial Communities in a Tropical Urban Waterway
Source: mSystems. 2018 Jun 5;3(3):e00136-17. doi: 10.1128/mSystems.00136-17 (PMC5989131; doi:10.1128/mSystems.00136-17)
Supplement: TABLE S1 [file sys003182236st1.docx]

| **Sample** | **Description** | **Total raw reads** | **After trimming*** | **#Reads mapped to NR** |
| --- | --- | --- | --- | --- |
| I3Pr1 | I_3 Pre, Event 1 | 90357002 | 88562109 | 18628348 |
| I2Pr2 | I_2 Pre, Event 2 | 48547378 | 47269125 | 19315766 |
| I1Po1 | I_1 Post, Event 1 | 39842076 | 37646011 | 20103251 |
| R5Po2 | R_5 Post, Event 2 | 50983274 | 49768179 | 20268339 |
| R6Po2 | R_6 Post, Event 2 | 58773496 | 57306384 | 21599178 |
| I4Pr1 | I_4 Pre, Event 1 | 49968752 | 48704985 | 21759790 |
| I2Po2 | I_2 Post, Event 2 | 54776318 | 53740858 | 21821911 |
| R6Po1 | R_6 Post, Event 1 | 51373612 | 48235604 | 21852395 |
| I5Pr2 | I_5 Pre, Event 2 | 53759154 | 51797175 | 23692592 |
| R3Pr2 | R_3 Pre, Event 2 | 59348424 | 57288299 | 24825411 |
| I1Po2 | I_1 Post, Event 2 | 55333032 | 53935711 | 25141637 |
| R4Pr2 | R_4 Pre, Event 2 | 58344740 | 57836218 | 25176241 |
| R2Pr2 | R_2 Pre, Event 2 | 56039048 | 53916614 | 25647651 |
| R1Po2 | R_1 Post, Event 2 | 58862236 | 57487503 | 25787787 |
| I6Pr2 | I_6 Pre, Event 2 | 54673098 | 53158348 | 25947719 |
| I5Po1 | I_5 Post, Event 1 | 58766808 | 57212209 | 26142142 |
| I1Pr2 | I_1 Pre, Event 2 | 63420900 | 61746688 | 26174531 |
| R3Po2 | R_3 Post, Event 2 | 56537990 | 55051917 | 26428151 |
| I5Po2 | I_5 Post, Event 2 | 62756700 | 60501606 | 26494115 |
| R3Pr1 | R_3 Pre, Event 1 | 53751618 | 52583114 | 26768180 |
| I6Pr1 | I_6 Pre, Event 1 | 62209932 | 60239045 | 26833543 |
| R1Pr2 | R_1 Pre, Event 2 | 55492266 | 54511084 | 26950512 |
| R1Pr1 | R_1 Pre, Event 1 | 54623638 | 53543634 | 27101256 |
| R2Po2 | R_2 Post, Event 2 | 58837350 | 57557891 | 27374143 |
| I4Po1 | I_4 Post, Event 1 | 58819336 | 56559083 | 27747776 |
| R4Po2 | R_4 Post, Event 2 | 63855194 | 62507402 | 27839047 |
| R4Po1 | R_4 Post, Event 1 | 56314242 | 54789224 | 28136957 |
| R4Pr1 | R_4 Pre, Event 1 | 54842234 | 53617775 | 28622778 |
| R2Pr1 | R_2 Pre, Event 1 | 59197562 | 58009566 | 28998787 |
| I3Po1 | I_3 Post, Event 1 | 58035090 | 55435693 | 29095714 |
| R3Po1 | R_3 Post, Event 1 | 58187258 | 56906595 | 29235138 |
| I5Pr1 | I_5 Pre, Event 1 | 62140938 | 60351189 | 29412316 |
| I4Pr2 | I_4 Pre, Event 2 | 67604772 | 65583452 | 29425902 |
| I6Po2 | I_6 Post, Event 2 | 70551282 | 67126034 | 30332942 |
| I1Pr1 | I_1 Pre, Event 1 | 69101700 | 67077630 | 33712316 |
| I3Po2 | I_3 Post, Event 2 | 78346742 | 76022042 | 34626616 |
| R5Po1 | R_5 Post, Event 1 | 72976700 | 70831584 | 34777695 |
| R6Pr2 | R_6 Pre, Event 2 | 85947028 | 83631120 | 36310363 |
| R5Pr2 | R_5 Pre, Event 2 | 84445328 | 82378893 | 36673222 |
| R2Po1 | R_2 Post, Event 1 | 55201042 | 53738822 | 36986787 |
| R1Po1 | R_1 Post, Event 1 | 74071192 | 72514832 | 38225086 |
| R6Pr1 | R_6 Pre, Event 1 | 78163312 | 76396896 | 39971330 |
| R5Pr1 | R_5 Pre, Event 1 | 77470290 | 75417446 | 40817210 |
| I3Pr2 | I_3 Pre, Event 2 | 102681142 | 99679389 | 46314603 |
| I2Pr1 | I_2 Pre, Event 1 | 99428428 | 97431861 | 46626390 |
| I2Po1 | I_2 Post, Event 1 | 98510094 | 95963585 | 49434240 |
| I4Po2 | I_4 Post, Event 2 | 66819284 | 64628560 | 52320189 |
| I6Po1 | I_6 Post, Event 1 | 89363150 | 87489914 | 62845659 |
