# Supplementary material for: Metagenomics Reveals the Influence of Land Use and Rain on the Benthic Microbial Communities in a Tropical Urban Waterway
Source: mSystems. 2018 Jun 5;3(3):e00136-17. doi: 10.1128/mSystems.00136-17 (PMC5989131; doi:10.1128/mSystems.00136-17)
Supplement: TABLE S2 [file sys003182236st2.docx]

A.

| **Cluster 1 (Res)** | **Cluster 1 (Ind)** | **Cluster 2** | **Cluster 3** | **Cluster 4** |
| --- | --- | --- | --- | --- |
| Nitrosovibrio | Thermosynechococcus | Asfivirus | Erythrobacter | Nitrosopumilus |
| Pimelobacter | Beggiatoa | Phaeovirus | Granulibacter | Candidatus Nitrosoarchaeum |
| Exiguobacterium | Dolichospermum | Alloprevotella | Parvularcula | Cenarchaeum |
| Pseudonocardia | Microchaete | Zunongwangia | Phycisphaera | Candidatus Glomeribacter |
| Janibacter | Saccharothrix | Porphyromonas | Acidiphilium | Laribacter |
| Nocardioides | Sulfuritalea | Joostella | Yualikevirus | Chromobacterium |
| Truepera | Microcoleus | Ornithobacterium | Hyphomonas | Pseudogulbenkiania |
| Aeromicrobium | Synechococcus | Flammeovirga | Maricaulis | Herbaspirillum |
| Nesterenkonia | Anabaena | Weeksella | Oceanicaulis | Magnetospirillum |
| Microbacterium | Nostoc | Methanobrevibacter | Frateuria | Advenella |

B.

| **Cluster 1** | **Cluster 2** |
| --- | --- |
| Flavonoid biosynthesis | Staphylococcus aureus infection |
| Isoflavonoid biosynthesis | Cytokine-cytokine receptor interaction |
| Caffeine metabolism | Salmonella infection |
| Carotenoid biosynthesis | Vibrio cholerae infection |
| Legionellosis | Bacterial invasion of epithelial cells |
| D-Arginine and D-ornithine metabolism | Influenza A |
| Stilbenoid, diarylheptanoid and gingerol biosynthesis | Pathogenic Escherichia coli infection |
| Phosphonate and phosphinate metabolism | Shigellosis |
| Bacterial secretion system | Glycosylphosphatidylinositol(GPI)-anchor biosynthesis |
| Xylene degradation | Complement and coagulation cascades |
