# Supplementary material for: Metagenomics Reveals the Influence of Land Use and Rain on the Benthic Microbial Communities in a Tropical Urban Waterway
Source: mSystems. 2018 Jun 5;3(3):e00136-17. doi: 10.1128/mSystems.00136-17 (PMC5989131; doi:10.1128/mSystems.00136-17)
Supplement: TABLE S3 [file sys003182236st3.docx]

A B

| *Source* | *df* | *MS* | *pseudo-F* | *p(perm.)* | *MS* | *pseudo-F* | *p(perm.)* |
| --- | --- | --- | --- | --- | --- | --- | --- |
| *LU* | *1* | 14954 | 2.28 | 0.260 | *83* | *2.51* | *0.504* |
| *B/A* | *1* | 5346 | 0.85 | 0.499 | *24* | *1.23* | *0.340* |
| *RE* | *1* | 8555 | 2.55 | **0.000** | *38* | *3.23* | ***0.008*** |
| *LU x B/A* | *1* | 4563 | 0.88 | 0.535 | *11* | *0.94* | *0.430* |
| *LU x RE* | *1* | 6570 | 1.96 | **0.005** | *33* | *2.80* | ***0.021*** |
| *B/A x RE* | *1* | 6263 | 1.87 | **0.007** | *20* | *1.66* | *0.129* |
| *LU x B/A x RE* | *1* | 5157 | 1.54 | **0.036** | *12* |  |  |
| *Residual* | *40* |  |  |  | *12* |  |  |
| *Pairwise tests* |  | ***RE1: B≠A****; RE2: B=A*  ***RE1: R≠I; RE2: R≠I*** | | | *Both RE: I ≠ R* | | |
