# Supplementary material for: Metagenomics Reveals the Influence of Land Use and Rain on the Benthic Microbial Communities in a Tropical Urban Waterway
Source: mSystems. 2018 Jun 5;3(3):e00136-17. doi: 10.1128/mSystems.00136-17 (PMC5989131; doi:10.1128/mSystems.00136-17)
Supplement: TABLE S4 [file sys003182236st4.docx]

|  |  | Land-use type | | Before *vs* After rain | |
| --- | --- | --- | --- | --- | --- |
| 1. Taxa | Measure | *F*_1,46_ | *p*(perm.) | *F*_1,46_ | *p*(perm.) |
|  | sqrt-BC | 7.6 | **<0.01** | 1.0 | 0.48 |
|  | Jaccard | 0.5 | 0.56 | 0.1 | 0.77 |

| 1. Functions | Level | *F_1,41_* | *p(perm.)* | *F_1,41_* | *p(perm.)* |
| --- | --- | --- | --- | --- | --- |
|  | *2* | *0.9* | *0.40* | *0.1* | *0.78* |
|  | *3* | *2.9* | *0.13* | *0.6* | *0.50* |
