# Supplementary material for: Metagenomics Reveals the Influence of Land Use and Rain on the Benthic Microbial Communities in a Tropical Urban Waterway
Source: mSystems. 2018 Jun 5;3(3):e00136-17. doi: 10.1128/mSystems.00136-17 (PMC5989131; doi:10.1128/mSystems.00136-17)
Supplement: TABLE S5 [file sys003182236st5.docx]

|  | *Bacteria and Archaea* | *All taxa* |
| --- | --- | --- |
| *SEED 2* | *0.68* | *0.72* |
| *SEED 3* | *0.85* | *0.86* |
