# Supplementary material for: Metagenomics Reveals the Influence of Land Use and Rain on the Benthic Microbial Communities in a Tropical Urban Waterway
Source: mSystems. 2018 Jun 5;3(3):e00136-17. doi: 10.1128/mSystems.00136-17 (PMC5989131; doi:10.1128/mSystems.00136-17)
Supplement: TEXT S1 [file sys003182236s1.docx]

**Diversity Indices**

*Shannon's Diversity Index:* This index measures diversity taking both, number of individuals and number of taxa, present in the community.

Formula used: $H=\sum_{i=0}^{i} \frac{n_{i}}{n}ln\frac{n_{i}}{n}$

Where $\frac{n_{i}}{n}$ is the propportion of *i^th^* taxa in the whole community.

*Buzas and Gibson's evenness:* This index measures the equitable distribution of taxa groups within the community.

Formula used: $E= \frac{e^{H}}{N}$ Where *N* is the number of taxa.
